# Feasibility, safety, and outcomes of a stratified fast-track care trajectory in pituitary surgery

**DOI:** 10.1007/s12020-020-02308-2

**Published:** 2020-05-02

**Authors:** Daniel J. Lobatto, Thea P. M. Vliet Vlieland, Wilbert B. van den Hout, Friso de Vries, Anne F. de Vries, Pieter J. Schutte, Marco J. T. Verstegen, Alberto M. Pereira, Wilco C. Peul, Nienke R. Biermasz, Wouter R. van Furth

**Affiliations:** 1 Center for Endocrine Tumors Leiden, Leiden University Medical Center, Leiden, The Netherlands; 2 Department of Neurosurgery, Leiden University Medical Center, Leiden, The Netherlands; 3 Department of Orthopaedics, Rehabilitation Medicine and Physical Therapy, Leiden University Medical Center, Leiden, The Netherlands; 4 Medical Decision Making, Department of Biomedical Data Sciences, Leiden University Medical Center, Leiden, The Netherlands; 5 Department of Medicine, Division of Endocrinology, Leiden University Medical Center, Leiden, The Netherlands; 6 Department of Neurosurgery, Haaglanden Medical Center, The Hague, The Netherlands

**Keywords:** Pituitary adenoma, Fast-track care trajectory, Feasibility and safety, Value Based Health Care, Endoscopic transsphenoidal surgery

## Abstract

**Objective:**

Discharge policies concerning hospitalization after endoscopic pituitary tumor surgery are highly variable. A few studies support fast-track discharge; however, this is not commonplace. Our goal was to report the transition to and evaluate the feasibility, safety, clinical- and patient-reported outcomes and costs of fast-track care in pituitary surgery.

**Methods:**

This observational study included 155 patients undergoing pituitary surgery between December 2016 and December 2018. Fast-track care consisted of planned discharge 2–3 days after surgery, followed by daily surveillance by a case manager. All outcomes were compared with patients not eligible for fast-track discharge. The total group (fast-track and non-fast-track) was compared with historic controls (*N* = 307).

**Results:**

A total of 79/155 patients (51%) were considered eligible for fast-track discharge, of whom 69 (87%) were discharged within 3 days. The total group was discharged more often within 3 days compared with historic controls (49 vs. 20%, *p* < 0.001), the total length of stay did not differ (5.3 vs. 5.7 days, *p* = 0.363). Although the total group had more readmissions compared with historic controls (17 vs. 10%, *p* = 0.002), no life-threatening complications occurred after discharge. On average, clinical- and patient-reported outcomes improved over time, both in the fast-track and non-fast-track groups. The mean overall costs within 30 days after surgery did not differ between the total group € 9992 (SD € 4562) and historic controls € 9818 (SD € 3488) (*p* = 0.649).

**Conclusion:**

A stratified fast-track care trajectory with enhanced postoperative outpatient surveillance after pituitary tumor surgery is safe and feasible. As expected, costs of the fast-track were lower than the non-fast-track group, however we could not prove overall cost-effectiveness compared with the historic controls.

## Introduction

Transsphenoidal surgery is the primary treatment option for most pituitary tumors [1–4] and over the past one to two decades the surgical technique of this procedure has shifted from a microscopic to an endoscopic approach in many centers [5], with reduced complication rates [6–8]. Careful monitoring of potential neurosurgical and endocrine complications is key, since they may still occur even in uneventful surgery. Patients remain in-hospital mainly for the monitoring of water and electrolyte imbalances caused by diabetes insipidus (DI) and/or delayed hyponatremia. Importantly, patients remain at risk for delayed hyponatremia, the primary reason for readmissions, for up to 14 days after pituitary tumor surgery [9, 10]. Effective management of postoperative water and electrolyte disturbances and awareness of hyponatremia symptoms is one of the main clinical challenges after pituitary tumor surgery both at an in- and outpatient setting [11].

In line with trends in general surgical care, fast-track care trajectories are applied in some centers that treat pituitary tumors. Common practice, however, is highly variable and many centers keep patients admitted for 5–8 days after uneventful surgery. The results from a limited number of studies support the concept that early discharge, e.g. discharge 2–3 days postoperatively, is feasible and safe [12–14]. However, sample sizes in the available studies were small (*N* < 50) and the occurrence of water and electrolyte disturbances during the immediate postdischarge period, as well as patients’ experiences were not evaluated. Length of stay (LOS) is an important measure, however, it is insufficient by itself to measure success of the surgery and studies should encompass patient-relevant outcomes [15, 16]. Furthermore, there is limited data on how to transition towards a fast-track discharge care trajectory, e.g. how to stratify patients regarding estimated date of discharge beforehand, how to perform home monitoring, and when to reconsider scheduled discharge.

In our tertiary referral center, part of the endoERN reference network, the general policy was to discharge patients 5 days after pituitary tumor surgery and we did not stratify patients on anticipated LOS. Through an innovation project we introduced a fast-track protocol with such a preoperative stratification and with daily outpatient monitoring after discharge. This predefined protocol was based on a literature-based risk evaluation [17]. The aim of the present study was to systematically and comprehensively evaluate the feasibility, safety, patient perspective, and costs of this fast-track care in pituitary tumor surgery, including pre- and postoperative risk assessments of potential complications. Results from this evaluation will provide important information for healthcare providers considering short-stay after surgery, which is necessary for expectation management surrounding the perioperative care trajectory.

## Methods

### Study design

This prospective cohort study was performed among a consecutive group of pituitary tumor patients treated endoscopically between December 2016 and December 2018 in a tertiary reference center. There were two reference groups: the first consisted of all pituitary tumor patients operated in the same period but were not considered eligible for fast-track discharge; the second was a retrospective cohort consisting of patients treated endoscopically prior to the intervention between January 2010 and November 2016 (historic controls). The Ethical Committee of the Leiden University Medical Center approved the prospective part prior to the study (p16.091). Consent was obtained from each patient after full explanation of the purpose and nature of all procedures used. For the historic control group, the same ethical committee approved a waiver of medical ethical review (G19.011).

### Study population

All patients were diagnosed with a pituitary tumor and underwent endoscopic transsphenoidal resection between January 2010 and December 2018 at our tertiary referral center, the Leiden University Medical Center in the Netherlands. From December 2016 onwards, patients were preoperatively assessed for eligibility for fast-track discharge. The systematic assessments were based according to a literature-based clinical protocol during a weekly pituitary multidisciplinary team meeting. Predefined reasons for ineligibility for the fast-track group were: need for emergency surgery (e.g. apoplexy), Cushing’s disease (CD), giant adenoma, craniopharyngioma, living far from the hospital, inadequate support network, and/or cognitive deficits. Directly after surgery, re-evaluation of the eligibility for fast-track discharge as well as an estimation of complication risks was performed by the treating neurosurgeon. Discharge was based on clinical grounds and only when deemed safe by the treating physician. This was re-assessed on a daily basis after surgery. Patients in the historic control group received care as usual.

### Interventions: fast-track care trajectory and usual care

Patients considered eligible for the fast-track care trajectory were instructed to actively participate in their own postoperative care by means of a standardized checklist which they had to report to the case manager on a daily basis after discharge. This checklist was composed to support patients to keep track of their fluid balance, weight, and relevant clinical signs and symptoms (Supplementary Table 1). Patients were instructed to report results digitally during the first 10–14 days after surgery. Those not capable of complying with our electronic surveillance were monitored through telephone consultation. The duration of the surveillance was dependent on the clinical judgment of the case manager and could be extended if deemed clinically necessary. Patients not eligible for fast-track discharge received care as usual up to December 2017, but along the way were also included in the outpatient monitoring after discharge. The surgical procedure has previously been published and was in line with existing guidelines [18–20]. All patients received low-dose perioperative corticosteroids (hydrocortisone) until postoperative confirmation of adequate pituitary–adrenal axis function was performed through dynamic testing or a fasting cortisol. Postoperative sodium levels were determined on POD7 for all patients and/or in case of symptoms of hyponatremia.

### Assessments

All data, with the exception of the prediction of complications, were obtained in the context of routine care and gathered by means of review of the medical records and questionnaires. Questionnaires could be filled in either digitally or on paper, both shown to provide equivalent results [21]. The treating neurosurgeon was asked to report his assessment on a case report form, directly after surgery.

### Disease-specific and sociodemographic characteristics

These included age, sex, comorbidities, tumor type, date of diagnosis, pituitary function, visual functioning, and cerebral nerve deficits. Comorbidities were categorized into diabetes mellitus, neurovascular, cardiovascular, pulmonary, ophthalmologic disease, or malignancies. Tumor types included: nonfunctioning pituitary adenoma, acromegaly (ACRO), CD, prolactinoma (PRL), TSH-producing adenoma, Rathke’s cleft cyst, or craniopharyngioma (Cranio). Pituitary function was defined as: (1) no deficits, (2) single hormone deficiency, (3) single hormone deficiency plus DI, (4) multiple hormone deficiencies, (5) multiple hormone deficiencies plus DI, and (6) DI alone. Visual functioning was defined as the presence of visual field deficits, or not. Prior treatments were described as: (1) no treatment, (2) prior medical (tumor) treatment, (3) prior surgery, and (4) prior radiotherapy.

### Outcome parameters

Primary outcomes were feasibility, safety, ability to predict postoperative complications, patient-reported experience, and costs. Patient-reported outcomes were secondary outcomes.

### Feasibility

Feasibility was defined as the proportion of patients allocated to the fast-track group, who were discharged 2–3 days after surgery and not readmitted within the fifth postoperative day (POD), which was often the date of discharge prior to the implementation of the protocol. Furthermore, adherence to the fast-track surveillance protocol was registered by means of the length of surveillance and the frequency and duration of fluid balance interventions.

### Safety

Safety was defined as the occurrence of a severe complication after discharge (Clavien–Dindo grade III or higher) [22]. Complications of interest were readmission within 30 days (general), transient DI/permanent DI/delayed hyponatremia/new pituitary deficiencies (endocrine complications) and postoperative CSF leak/epistaxis/intracranial hemorrhage (neurosurgical complications). Transient DI was defined as necessity of treatment (desmopressin) up to 6 months after surgery. Permanent DI was defined as treatment for more than 6 months. CSF leaks during surgery with prompt closure were not considered a postoperative complication and were not a contraindication for early discharge. For readmissions, the primary reason of readmission, duration of readmission in days, and postoperative date of readmission were recorded.

### Ability to predict postoperative complications

The estimated risk of complications was evaluated immediately after surgery by the neurosurgeons to investigate whether this would help to differentiate between patients at risk of complications and those who were not. The likelihood of complications included transient DI, permanent DI, new onset of pituitary deficiencies, epistaxis, postoperative CSF leak, and intracranial hemorrhage. The likelihood of complications was dichotomized into not likely and possible, from which the sensitivity and specificity were calculated.

### Patient-reported outcome measures (PROMs)

A comprehensive set of PROMs was administered at baseline (preoperatively) and 6 weeks after surgery. Changes in PROMs were calculated as between group differences corrected for baseline. Disease bother was measured through the Leiden Bother and Needs Questionnaire-pituitary (LBNQ-pituitary) [23], which was modified in order to make it suitable for perioperative repeated measurements. The total score ranges from 0 to 100, with higher scores indicating a greater disease bother or need for help. Health-related quality of life (HRQoL) was measured using the short form-36, from which physical and mental component scores can be calculated. These range from 0 to 100, with higher scores indicating better HRQoL [24]. Health status was assessed using the five-level EQ-5D index (Dutch tariff, anchored at 0 (as bad as death) and 1 (perfect health)), and the EQ-5D VAS (ranging from 0 to 100) [25, 26]. Higher scores indicate a better perceived health status. Visual functioning was assessed through the visual functioning questionnaire-25 (range 0 to 100), and higher scores indicate better visual functioning [27].

### Patient-reported experience measures (PREMs)

Patient-reported experiences were measured 4 weeks after surgery among patients in the fast-track group by means of a self-designed questionnaire and included experience of delivered care, sense of safety at home during the first 3 days at home, as well as the period after (day 4 through day 7). This questionnaire also assessed the self-perceived patient empowerment on a five-point Likert scale (range: “not at all” to “completely”) and the self-perceived optimal discharge date (range: −2 to +4 days).

### Costs

Costs were estimated from a healthcare perspective, at price level 2019. Hospital care included the initial admission (regardless of duration) and all subsequent hospital care up to 30 days after surgery (including readmission, emergency room visits, outpatient clinic visits, e-mail, and telephone contacts). All healthcare use was assessed from patient records, except for outpatient clinic visits in the non-fast-track and historic cohort, which was set at two visits, unless hospitalization lasted for more than 30 days. Costs for surgery were derived from the Dutch Healthcare Authority [28, 29], and all other costs from Dutch reference prices designed to standardize economic evaluations (Supplementary Table 2) [30].

### Statistical analysis

All statistical analyses were performed with SPSS 25.0 software (SPSS Inc., Armonk, NY, USA). Nominal variables are presented as frequencies with percentages, numerical variables as means and standard deviations (SD), or medians with interquartile ranges (IQR). Comparisons were made between the fast-track and non-fast-track groups, as well as between the historic group and the total group (fast-track and non-fast-track). Comparisons were performed through one-way ANOVA, Chi-square analyses, Fisher’s exact test, or general linear mixed models (GLM), where applicable. The sensitivity (Se) and specificity (Sp) were used to calculate the discriminative ability of the predictions, as approximated by ½(Se + Sp) [31]. Longitudinal analysis was performed via GLM analysis and results are presented as means with corresponding standard errors. For all analyses, the level of significance was set at *p* < 0.05 (two-sided). Missing data on the validated questionnaires were handled by parcel summary imputation [32].

The historic control group comprised of all patients surgically treated between January 2010 and December 2016, including those with diagnoses that were not considered eligible for fast-track surgery. In an attempt to compare the fast-track group with representative patients from our historical cohort, all comparisons were repeated after exclusion of patients with CD, Cranio, giant adenomas, and acute apoplexy (sensitivity analysis).

## Results

Between December 2016 and December 2018, a total of 155 patients were surgically treated for a pituitary tumor. Patients had a mean age of 48.4 years (SD 16.9) and 54% were female. Most patients had an NFA (45%), followed by ACRO and PRL (both 16%), CD (14%), and other tumors (9%). Among the historic cohort, surgical treatment was performed among 307 patients, with a mean age of 51.5 years (SD 16.9). Of these, 53% were female and most patients also had an NFA (45%), followed by CD (17%), ACRO (16%), PRL (10%), and other tumors (12%) (Table 1).

**Table 1:** Baseline characteristics of patients with a pituitary tumor

	Fast-track (*N* = 79)	Non-fast-track (*N* = 76)	*p* value*	Total (*N* = 155)	Historic cohort (*N* = 307)	*p* value**
Sociodemographic characteristics						
Female gender, *N* (%)	43 (54.4)	40 (52.6)	0.873	83 (53.5)	163 (53.1)	1.000
Age in years, mean (SD)	47.2 (16.0)	49.7 (17.9)	0.368	48.4 (16.9)	51.5 (16.9)	0.069
Comorbidities, *N* (%)						
Diabetes mellitus	3 (3.8)	12 (15.8)	**0.014**	15 (9.7)	38 (12.4)	0.442
Neurovascular disease	11 (13.9)	10 (13.2)	1.000	21 (13.5)	36 (11.7)	0.653
Cardiovascular disease	17 (21.5)	40 (52.6)	**<0.001**	57 (36.8)	121 (39.4)	0.614
Malignancies	8 (10.1)	3 (3.9)	0.211	11 (7.1)	33 (10.7)	0.242
Pulmonary disease	1 (1.3)	5 (6.6)	0.112	6 (3.9)	27 (8.8)	0.057
Ophthalmologic disease	15 (19.0)	15 (19.7)	1.000	30 (19.4)	55 (17.9)	0.705
Disease-specific characteristics						
Tumor type, *N* (%)						
NFA	40 (50.6)	30 (39.5)		70 (45.2)	137 (44.6)	
ACRO	15 (19.0)	10 (13.2)		25 (16.1)	50 (16.3)	
CD	0 (0.0)	21 (27.6)		21 (13.5)	53 (17.3)	
PRL	20 (25.3)	5 (6.6)		25 (16.1)	30 (9.8)	
RCC	4 (5.1)	0 (0.0)		4 (2.6)	13 (4.2)	
Cranio	0 (0.0)	9 (11.8)		9 (5.8)	19 (6.2)	
TSH-oma	0 (0.0)	1 (1.3)	**<0.001**	1 (0.6)	5 (1.6)	0.433
Tumor size, *N* (%)						
Micro	19 (24.1)	20 (26.3)		39 (25.2)	64 (20.8)	
Macro	60 (75.9)	45 (59.2)		105 (67.7)	226 (73.9)	
Giant	0 (0.0)	11 (14.5)	**0.001**	11 (7.1)	16 (5.2)	0.374
Cavernous sinus invasion, *N* (%)	12 (15.2)	11 (14.5)	1.000	23 (14.8)	72 (23.5)	**0.041**
Time since diagnosis in years, median (IQR)	1.1 (0.2–5.2)	0.2 (0.0–1.7)	**0.002**	0.4 (0.1–3.9)	0.5 (0.1–3.6)	0.483
Prior treatments, *N* (%)						
No treatment	40 (50.6)	45 (59.2)	0.334	85 (54.8)	185 (60.3)	0.273
Medication	31 (39.2)	25 (32.9)	0.504	56 (36.1)	75 (24.4)	**0.009**
Surgery	11 (13.9)	15 (19.7)	0.393	26 (16.8)	62 (20.2)	0.384
Radiotherapy	0 (0.0)	1 (1.3)	0.490	1 (0.6)	7 (2.3)	0.277
Apoplexy, *N* (%)	4 (5.1)	16 (21.1)	**0.004**	20 (12.9)	24 (7.8)	0.093
Preoperative endocrine status, *N* (%)						
No deficits	40 (50.6)	33 (44.0)		73 (47.4)	152 (49.8)	
Single hormone deficiency	15 (19.0)	10 (13.3)		25 (16.2)	34 (11.1)	
Single hormone deficiency + DI	0 (0.0)	1 (1.3)		1 (0.6)	0 (0.0)	
Multiple hormone deficiencies	24 (30.4)	28 (37.3)		52 (33.8)	118 (38.7)	
Multiple hormone deficiencies + DI	0 (0.0)	2 (2.7)		2 (1.3)	1 (0.3)	
DI alone	0 (0.0)	1 (1.3)	0.308	1 (0.6)	0 (0.0)	0.095
Preoperative visual status, *N* (%)						
No deficits	51 (64.6)	32 (42.1)	**0.041**	83 (53.5)	159 (52.0)	1.000
Cranial nerve palsy, *N* (%)	2 (2.5)	10 (13.2)	**0.017**	12 (7.7)	13 (4.2)	0.128
Completed questionnaire, *N* (%)	65 (82.3)	42 (55.3)		107 (69.0)	16 (5.2)	

Due to rounding, not all percentages of the categorical variables add up to 100%

*N* number, *SD* standard deviation, *IQR* interquartile range, *NFA* nonfunctioning pituitary adenoma, *ACRO* acromegaly, *CD* Cushing’s disease, *PRL* prolactinoma, *RCC* Rathke’s cleft cyst, *Cranio* craniopharyngioma, *TSH* thyroid-stimulating hormone

Bold values indicate statistical significance *p* < 0.05

*Fast-track vs. non-fast-track; **Total vs. historic cohort

### Feasibility

Of the 155 patients, 79 patients (51%) were preoperatively considered eligible for fast-track discharge. Of these, 69 patients (87%) were discharged 2–3 days after surgery as planned (POD2: *N* = 37, POD3: *N* = 32) and three of these patients (4%) needed to be readmitted within POD5. Among the patients eligible for fast-track discharge that required a stay of more than 3 days (range 4–17 days), one was readmitted within the fifth POD. In addition, among patients not considered eligible at preoperative counseling, 7 (9%) were successfully discharged after 2–3 days after surgery (POD2: *N* = 1, POD3: *N* = 6). In comparison, in the historic cohort, only 61 patients (20%) were discharged 2–3 days after surgery (POD2: *N* = 17, POD3: *N* = 44).

**Fig. 1:**
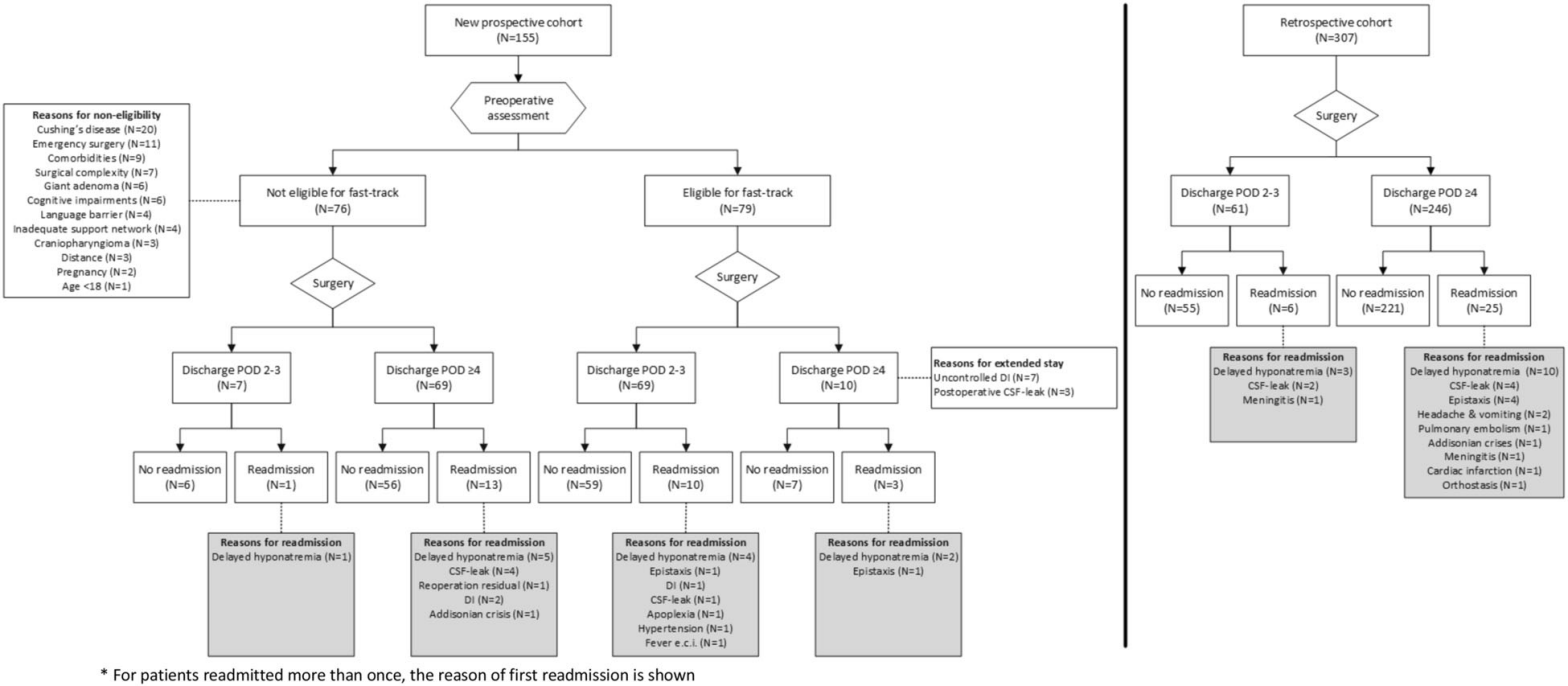
Flow-chart of patients surgically treated for a pituitary tumor

**Fig. 2:**
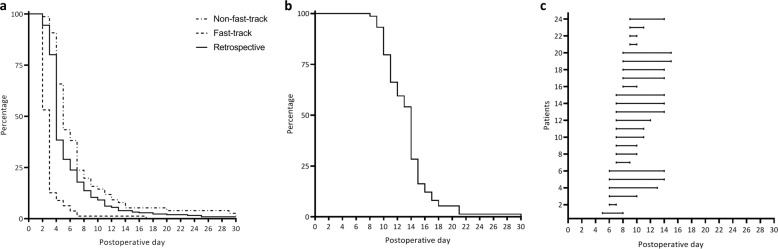
Survival curve of duration of date of discharge (**a**); active surveillance after surgery (**b**); onset and duration of fluid restrictions per patient (**c**)

Among patients in the fast-track group, reasons for delaying discharge were uncontrolled DI in seven patients (9%) and a postoperative CSF leak in three patients (4%). The three most frequent reasons for a priori non-eligibility were CD (*N* = 20, 26%), emergency surgery (*N* = 11, 14%), and due to various comorbidities (*N* = 10, 13%) (Figs. 1 and 2a).

Adherence to the fast-track surveillance protocol is depicted in Fig. 2. Surveillance by the case manager was stopped on average on POD14 (IQR 11–15) (Fig. 2b). Reasons for extending the period of surveillance beyond the initially planned 14 days were a prescheduled sodium check on POD15 (*N* = 8), fluctuating fluid balance/uncontrolled DI (*N* = 9), persisting physical complaints (*N* = 2), and a previous readmission (*N* = 2). During follow-up, 24 patients received a fluid restriction, which started on average 7.3 days (SD 1.1) after surgery and lasted for a mean of 4.6 days (SD 2.4) (Fig. 2c). All but five patients were able to provide daily evaluations digitally and were monitored through telephone consultation.

### Length of stay

Patients in the fast-track group had a significantly shorter LOS compared with the non-fast-track group (3.0 vs. 7.6 days, *p* < 0.001), however the overall LOS of the total group was not significantly lower compared with the historic cohort (5.3 vs. 5.7 days, *p* = 0.363) (Table 2).

**Table 2:** Surgical outcomes and costs among 462 surgically treated patients with a pituitary tumor stratified according to cohort

	Fast-track (*N* = 79)	Non-fast-track (*N* = 76)	*p* value*	Total (*N* = 155)	Historic cohort (*N* = 307)	*p* value**
Length of stay, mean (SD)	3.0 (1.9)	7.6 (8.6)	**<0.001**	5.3 (6.6)	5.7 (4.9)	0.363
Complications						
Number of readmitted patients, *N* (%)	13 (16.5)	14 (18.4)	0.747	27 (17.2)	31 (10.1)	**0.025**
Length of stay of all readmissions, mean (SD)	3.6 (2.7)	4.3 (4.2)	0.647	4.0 (3.5)	4.1 (3.4)	0.917
Any complication, *N* (%)	38 (48.1)	46 (60.5)	0.147	84 (54.2)	179 (58.3)	0.427
Transient DI, *N* (%)	20 (25.3)	28 (36.8)	0.165	48 (31.0)	53 (17.3)	**0.001**
Permanent DI, *N* (%)	3 (3.8)	7 (9.2)	0.204	10 (6.5)	17 (5.5)	0.834
Delayed hyponatremia, *N* (%)	9 (11.4)	14 (18.4)	0.262	23 (14.8)	31 (10.1)	0.167
New onset pituitary deficiency, *N* (%)	4 (5.1)	9 (11.8)	0.148	13 (8.4)	22 (7.2)	0.709
Postoperative CSF leak, *N* (%)	3 (3.8)	8 (10.5)	0.126	11 (7.1)	25 (8.2)	0.719
Epistaxis, *N* (%)	10 (12.7)	2 (2.6)	**0.032**	12 (7.7)	29 (9.4)	0.606
Postoperative intracranial hemorrhage, *N* (%)	0 (0.0)	1 (1.3)	0.490	1 (0.6)	3 (1.0)	1.000
Hospital costs (in euro’s)						
Admission, mean (SD)	7249 (1318)	10394 (5868)	**<0.001**	8791 (4488)	9127 (3306)	0.363
Readmission, mean (SD)	438 (1221)	608 (1759)	0.486	521 (1506)	323 (1287)	0.141
Emergency room visits, mean (SD)	42 (108)	50 (153)	0.677	46 (132)	25 (85)	**0.039**
Outpatient clinic visits, mean (SD)	55 (102)	332 (68)	**<0.001**	191 (164)	343 (34)	**<0.001**
E-mail contacts, mean (SD)	626 (284)	0 (0)	**<0.001**	319 (373)	0 (0)	**<0.001**
Telephone contacts, mean (SD)	242 (184)	0 (0)	**<0.001**	123 (178)	0 (0)	**<0.001**
Total hospital costs, mean (SD)	8652 (1748)	11,384 (5974)	**<0.001**	9992 (4562)	9818 (3488)	0.649

*N* number, *SD* standard deviation, *IQR* interquartile range, *DI* diabetes insipidus, *CSF* cerebrospinal fluid

Bold values indicate statistical significance *p* < 0.05

*Fast-track vs. non-fast-track; **Total vs. historic cohort

### Safety

**Fig. 3:**
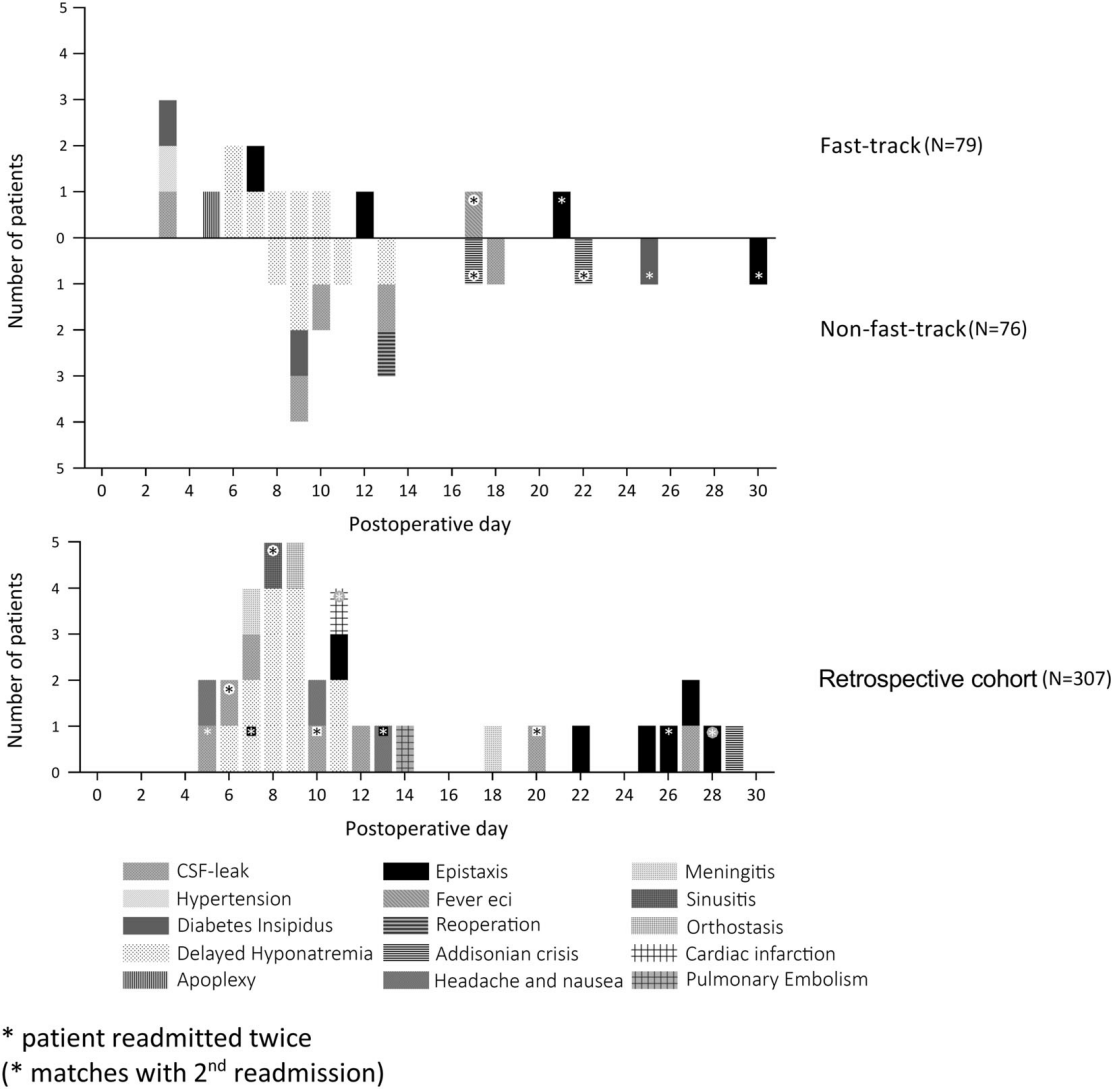
Readmissions per day and reasons for readmission among all patients

No life-threatening complications occurred after discharge (Clavien–Dindo grade IV), in particular not in the period between fast-track discharge and “regular” discharge. However, two patients (2.5%) were readmitted for the surgical treatment of an epistaxis late after fast-track discharge (grade III, POD12 and 21). In the fast-track discharge group, a total of 13 patients (16%) were readmitted after discharge, on average 8.5 days (SD 6.0) after surgery. This was most frequently due to delayed hyponatremia (*N* = 6, 43%) and did not differ with the non-fast-track group, among which 14 (18%) were readmitted (*p* = 0.747). Patients readmitted among the non-fast-track group were readmitted on average 13.2 days (6.2 SD) after surgery and also most frequently due to delayed hyponatremia (*N* = 6, 43%). In the historic cohort group, there were significantly fewer readmissions compared with the total group (*N* = 31, 10% vs. *N* = 27, 17%, *p* = 0.03). In the total group, the reason for readmission was most frequently due to delayed hyponatremia (*N* = 13, 42%) (Fig. 3, Table 2).

### Ability to predict postoperative complications

On average, the ability to predict complications after surgery was low. For all complications, a high specificity was combined with a low sensitivity or vice versa. The discriminative ability ranged from 45 to 62%, showing that it was difficult to predict which patients are at risk of complications after surgery (Table 3).

**Table 3:** Postoperative evaluation of complication risks by treating neurosurgeon among fast-track patients (*N* = 64)

	Total	Unlikely	Possible	Sensitivity	Specificity	Discriminative ability
Transient DI, *N*						
Yes	14	0	14			
No	50	12	38	100%	24%	62%
Permanent DI, *N*						
Yes	3	3	0			
No	61	54	7	0%	89%	44%
New onset pituitary deficiency, *N*						
Yes	4	2	2			
No	60	27	33	50%	45%	48%
CSF leak, *N*						
Yes	3	2	1			
No	61	39	22	33%	64%	49%
Epistaxis, *N*						
Yes	7	1	6			
No	55	19	36	86%	35%	60%
Postoperative hemorrhage, *N*						
Yes	0	0	0			
No	64	47	17	–	73%	–

*N* number, *DI* diabetes insipidus, *CSF* cerebrospinal fluid

### Patient-reported experience

Among the fast-track group, the overall patient satisfaction about the delivered care after discharge was a 7.9 (SD 1.5, scale 1–10), which was significantly lower among patients readmitted after discharge compared with those who were not readmitted (8.1 vs. 7.0, *p* = 0.04). The mean overall sense of safety at home during the first 3 days after discharge was 6.7 (SD 2.5, scale 1–10), which was not statistically different between patients who were not readmitted compared with those who were (mean 6.9 vs. 5.3, *p* = 0.08). After the initial period at home (3 days), the mean overall sense of safety improved to a mean of 7.7 (SD 1.8, scale 1–10), which was significantly lower among patients readmitted compared with those who were not (6.0 vs. 8.0, *p* = 0.001). Over half of the patients (54%) perceived themselves as very/completely empowered, which did not differ between both groups (*p* = 1.00). Nearly 40% of patients (*N* = 23/58), however, would have preferred to stay admitted one or more days longer, which was significantly higher among readmitted patients (*p* = 0.02) (Table 4). This was not assessed in the non-fast-track group, nor in the historical controls.

**Table 4:** Patient-perceived satisfaction, sense of safety, and perceived optimal discharge date among patients eligible for fast-track

	Total (*N* = 79)	No readmission (*N* = 69)	Readmission (*N* = 10)	*p* value
Completed questionnaire, *N* (%)	58 (73.4)	49 (71.0)	9 (90.0)	
Delivered care after discharge, mean (SD)	7.9 (1.5)	8.1 (1.5)	7.0 (1.4)	**0.044**
Sense of safety at home: day 1–3 (scale 1–10), mean (SD)	6.7 (2.5)	6.9 (2.4)	5.3 (2.9)	0.078
Sense of safety at home: after 3 days (scale 1–10), mean (SD)	7.7 (1.8)	8.0 (1.6)	6.0 (1.9)	**0.001**
Sense of self-empowerment, *N* (%)				
Not at all	1 (1.7)	1 (2.0)	0 (0.0)	
Slightly	3 (5.2)	3 (6.1)	0 (0.0)	
Moderately	22 (37.9)	18 (36.7)	4 (44.4)	
Very	16 (27.6)	13 (26.5)	3 (33.3)	
Completely	16 (27.6)	14 (28.6)	2 (22.2)	1.00
Patient-perceived optimal date of discharge, *N* (%)				
1 day earlier	3 (5.2)	3 (6.1)	0 (0.0)	
Exact the same day	32 (55.2)	29 (59.2)	3 (33.3)	
1 day later	8 (13.8)	8 (16.3)	0 (0.0)	
2 or more days later	15 (25.9)	9 (18.4)	6 (66.7)	**0.023**

*N* number, *SD* standard deviation, (bold) *p* < 0.05

### Costs

The mean costs of perioperative treatment were € 8652 (SD € 1748) for patients in the fast-track group, which was significantly lower compared with patients in the non-fast-track group (€ 11,384; SD € 5974, *p* < 0.001). There was no significant difference in costs between the total group (€ 9992; SD € 4562) and the historic cohort (€ 9818; SD € 3488, *p* = 0.649) (Table 2).

### Patient-reported outcomes

In general, the disease burden decreases among patients after surgery compared with prior to surgery; HRQoL improves, nasal morbidity decreases, and visual functioning improves after surgery irrespective of whether a patient is in the fast-track or non-fast-track group, nor were there any differences between the total group and the historic controls (Supplementary Table 3).

### Sensitivity analysis

Excluding patients with diagnoses not considered eligible for fast-track from the historic control group yielded a selection of 213 patients. Both groups (fast-track and selected historic cohort) were grossly comparable (Supplementary Table 4). Among the fast-track group, we found a shorter LOS (mean 3.0 vs. 5.1 days, *p* < 0.001), but higher occurrence of transient DI (25 vs. 15%, *p* = 0.036). The costs in the fast-track group were significantly lower than in the selected historic control group (€ 8652 vs. € 9266, *p* = 0.021) (Supplementary Table 5).

## Discussion

This study shows that fast-track discharge after pituitary surgery is feasible and can be safely implemented when incorporated in a well-defined care trajectory with stratification. For a select group of patients, we were able to decrease the overall LOS by including the patient as an active participant, while being under surveillance of a dedicated case manager. Early discharge was possible in 87% of preoperatively identified cases, and in an additional 9% of the non-eligible cases. It remains difficult, however, to adequately predict complications and readmissions and therefore we advocate that all patients require monitoring up to at least 14 days postoperatively.

After the reported evaluation period we implemented the described protocol in our practice. It is likely that with increasing experience, more patients can be stratified towards the fast-track discharge group. Restriction of early postoperative vasopressin use and earlier institution of fluid restrictions may reduce the number of readmissions. It is furthermore probably possible to reduce the number of contact moments without compromising patient safety.

Readmissions appeared relatively high (16%) in the fast-track discharge, as well as the non-fast-track discharge group (18%) compared with historical controls (10%). Since this was mainly due to SIADH and the protocol was directed to detect patients at risk at an early stage, it is likely that we were more aware of diagnosing and treating this complication at an early stage. Importantly, no life-threatening complications occurred in the home setting. So, we conclude that the high readmission rate most likely reflects the intense attention to postoperative complications combined with our low threshold for readmittance. Results shown in this study provide useful information that will facilitate better expectation management, improve water and electrolyte imbalance protocols, decrease the occurrence of delayed hyponatremia, and subsequent readmissions.

Even though there are more centers that discharge patients at POD2 or even sooner, the feasibility and safety has only been scarcely evaluated [12–14]. In the postoperative phase, the risk of delayed hyponatremia remains an important problem. This study confirms previous data that patients are at risk for readmission due to delayed hyponatremia, for which reported peak incidence ranges from POD4 to POD7 [10, 11, 33, 34]. Our study adds to this that late complications do occur even up to 30 days after surgery. Our study provides practical tips for those who consider the transition to fast-track care. It will allow a shift from inpatient general nursing care to an extended period of daily outpatient care by a specialized case manager, dedicated to treat both endocrinological and neurosurgical aspects.

One of the main reasons to initiate this fast-track protocol was the impression that our patients thought that postoperative hospitalization was only for complication surveillance, not for actual needed care. An unanticipated result is the lower than expected overall sense of safety at home during the first 3 days as perceived by patients discharged early after surgery. Control data of experienced safety in the first days after discharge are not available for pituitary surgery, so we do not know if this is uncommon or not. Furthermore, the majority of our patients indicated afterwards that they were content with the day of discharge. Based on obtained patients’ experiences, we doubt whether further shortening the admission period, albeit commonplace in some centers, would be desired by patients. Patients who were readmitted reported a lower perceived safety at home, which might be explained in part by the fact that patients who experience adverse events after discharge often have lower evaluations of care [35]. So, patient education, expectation management, and additional strategies to improve the sense of safety are warranted.

There are several recent publications regarding standardized fluid restrictions in the short-term postoperative phase [36–39]. Benefits from this standardized fluid restriction approach are the low-threshold of application of fluid restrictions and the specific targeting of patients at risk for delayed hyponatremia. Even though delayed hyponatremia is the most frequent reason for readmission, this approach is less suitable for management of other complications, since patients guided through a standardized fluid restriction protocol are not followed as strictly and the adaptability is lower compared with our fast-track protocol. The results from these fluid restriction studies, in combination with our fast-track results, might suggest that a combined approach, consisting of daily contact in combination with low-threshold fluid restrictions, should be considered. In this context, it is also important to consider a restriction of intraoperative fluids for early discrimination between DI and perioperative fluid overload.

The size, comprehensiveness, and prospective nature are the main strengths of this study. Previous studies had smaller sample sizes (up to 47 patients) compared with our study. By comparing results from the fast-track group with the non-fast-track group, as well as with the historic cohort group, we have provided more accurate reference data from which we drew our conclusions regarding the outcomes of our fast-track protocol. Ideally, we would have performed a randomized controlled trial or a cluster randomized trial, however due to the rarity of the disease, the heterogeneity of the population, and the odds of contamination of the non-surveillance group, these methods were deemed not feasible [40].

Also, by presenting and comparing the results of these three groups we have attempted to provide insight into the possible occurrence of selection bias. The differences in terms of costs appear to be small, but promising, if only those patients from the historic cohort are considered that meet eligibility criteria for fast-track. Nevertheless, results of the sensitivity analysis should be interpreted with caution, since it is unknown what direction the results of the historic cohort would go towards when the selection of patients would have been performed like that of the fast-track group. We potentially introduced recall bias, which was introduced by asking patients to recollect their initial sense of safety several weeks after discharge instead of on the actual date itself.

Another limitation of this study is the standardization of costs for the retrospective and non-fast-track group. This might have resulted in lower overall costs for these groups of patients and future research should focus more on the cost aspect of the intervention. Preferably this should be done through a time-driven-activity-based-costing approach, which is advocated within the model of value-based healthcare [41]. Also, further evaluation of optimal transition towards a fast-track care trajectory, as well as evaluation of differences between patient evaluations and how to optimally empower patients is necessary to optimize this care trajectory.

## Conclusion

Discharging selected patients 2–3 days after transsphenoidal surgery through a well-defined fast-track care trajectory appears feasible and safe. Although the overall costs of the fast-track group were lower compared with the non-fast-track group, the overall costs between the total group and the historic group did not differ, while a specialized case manager provided prolonged daily monitoring. Since the prediction of complications remains difficult and readmissions do occur, monitoring is needed also after uneventful surgery. With this approach we did not encounter any life-threatening situations by expediting the date of discharge in a large group of patients. Additional patient education and expectation management are needed to improve the reassurance about the safety of early discharge.

## Supplementary material

Supplementary Table 1

Supplementary Table 2

Supplementary Table 3

Supplementary Table 4

Supplementary Table 5
