# Supplementary material for: Feasibility, safety, and outcomes of a stratified fast-track care trajectory in pituitary surgery
Source: Endocrine. 2020 May 2;69(1):175–87. doi: 10.1007/s12020-020-02308-2 (PMC7343751; doi:10.1007/s12020-020-02308-2)
Supplement: Supplementary file 1 — Supplementary Table 1 [file 12020_2020_2308_MOESM1_ESM.docx]

Title: Feasibility, safety and outcomes of a stratified fast-track care trajectory in pituitary surgery

Journal: Endocrine

Authors: Daniel J. Lobatto^1,2^, Thea. P.M. Vliet Vlieland^1,3^, Wilbert B. van den Hout^1,4^, Friso de Vries^1,5^, Anne F. de Vries^1,2^, Pieter J. Schutte^1,2^, Marco J.T. Verstegen^1,2^, Alberto M. Pereira^1,5^, Wilco C. Peul^1,2,6^, Nienke R. Biermasz^1,5^, Wouter R. van Furth^1,2^

Affiliations: Center for Endocrine Tumors Leiden, Leiden University Medical Center, Leiden, The Netherlands^1^;

Department of Neurosurgery, Leiden University Medical Center, Leiden, The Netherlands^2^;

Department of Orthopaedics, Rehabilitation Medicine and Physical Therapy, Leiden University Medical Center, Leiden, The Netherlands^3^;

Medical Decision Making, Department of Biomedical Data Sciences, Leiden University Medical Center, Leiden, The Netherlands^4^;

Department of Medicine, Division of Endocrinology, Leiden University Medical Center, Leiden, The Netherlands^5^;

Department of Neurosurgery, Haaglanden Medical Center, The Hague, The Netherlands^6^

E-mail of Corresponding author: d.j.lobatto@lumc.nl

**Supplementary table 1.** Daily questionnaire after pituitary surgery

| 1. What was your fluid balance during the previous 24 hours? | |
| --- | --- |
| In: ml | Out: ml |

| 1. What was your weight after awakening this morning? |
| --- |
| Kilograms |

| 1. Did you have a fever during the previous 24 hours (temperature of >38.5^○^C)? | | |
| --- | --- | --- |
| □ | Yes | 🡪 Take a double dosage of hydrocortisone and contact the case manager |
| □ | No | 🡪 No further action required |

| 1. How are you feeling today? | | |
| --- | --- | --- |
| □ | Not good | 🡪 Fill in the rest of the questionnaire |
| □ | Reasonably well | 🡪 Fill in the rest of the questionnaire |
| □ | Good | 🡪 No further action required |

| 4b. How are you feeling compared to yesterday? | | |
| --- | --- | --- |
| □ | Worse compared to yesterday | 🡪 Fill in the rest of the questionnaire |
| □ | Unchanged | 🡪 Fill in the rest of the questionnaire |
| □ | Better compared to yesterday | 🡪 No further action required |
| □ | I don’t have any complaints | 🡪 No further action required |

| 1. Are you increasingly tired and/or are you feeling washed-out? | | |
| --- | --- | --- |
| □ Yes | 🡪 it’s getting worse | 🡪 Take a double dosage of hydrocortisone and contact the case manager |
|  | 🡪 it’s about the same | 🡪 No further action required |
|  | 🡪 it’s improving | 🡪 No further action required |
| □ No | 🡪 No further action required | |

| 1. Have you had complaints of nausea and/or did you vomit in the previous 24 hours? | |
| --- | --- |
| □ Yes | 🡪 Take a double dosage of hydrocortisone and contact the case manager |
| □ No | 🡪 No further action required |

| 1. Have you lost your appetite? | | |
| --- | --- | --- |
| □ Yes | 🡪 Worse compared to before | 🡪 Contact the case manager |
|  | 🡪 Unchanged | 🡪 No further action required |
|  | 🡪 Better compared to before | 🡪 No further action required |
| □ No | 🡪 No further action required | |

| 1. Do/Did you have a headache? | | | |
| --- | --- | --- | --- |
| □ Yes | 🡪 Worse compared to before | 🡪 Contact the case manager |  |
|  | 🡪 Unchanged | 🡪 No further action required |  |
|  | 🡪 Better compared to before | 🡪 No further action required |  |
| □ No | 🡪 No further action required | | |

| 1. Do you have any physical complaints at the moment? | | |
| --- | --- | --- |
| □ | Yes | 🡪 Please report your complaints |
| □ | No | 🡪 No further action required |

| 1. Do you have unquenchable thirst despite drinking? | |
| --- | --- |
| □ Yes | 🡪 Take a double dosage of hydrocortisone and contact the case manager |
| □ No | 🡪 No further action required |

| 1. Do you feel like you have to urinate more than usual? | |
| --- | --- |
| □ Yes | 🡪 Contact the case manager |
| □ No | 🡪 No further action required |

| 1. Is there leakage of fluid out of your nose (not: old blood or fluid from the nasal douche)? | |
| --- | --- |
| □ Yes | 🡪 Contact the case manager |
| □ No | 🡪 No further action required |

| 1. How is your vision since the surgery? | |
| --- | --- |
| □ It’s getting worse | 🡪 Contact the case manager |
| □ Unchanged | 🡪 No further action required |
| □ It’s improving | 🡪 No further action required |

🡪 In case all questions have resulted in the answer “No further action required” we request you to send your answers to the case manager before 10:00 a.m.

🡪 In case of a fever, severe fatigue, nausea, vomiting, we urge you to take a double dosage of hydrocortisone and contact the case manager or on-call endocrinologist immediately.
