# Supplementary material for: Feasibility, safety, and outcomes of a stratified fast-track care trajectory in pituitary surgery
Source: Endocrine. 2020 May 2;69(1):175–87. doi: 10.1007/s12020-020-02308-2 (PMC7343751; doi:10.1007/s12020-020-02308-2)
Supplement: Supplementary file 2 — Supplementary Table 2 [file 12020_2020_2308_MOESM2_ESM.docx]

Title: Feasibility, safety and outcomes of a stratified fast-track care trajectory in pituitary surgery

Journal: Endocrine

Authors: Daniel J. Lobatto^1,2^, Thea. P.M. Vliet Vlieland^1,3^, Wilbert B. van den Hout^1,4^, Friso de Vries^1,5^, Anne F. de Vries^1,2^, Pieter J. Schutte^1,2^, Marco J.T. Verstegen^1,2^, Alberto M. Pereira^1,5^, Wilco C. Peul^1,2,6^, Nienke R. Biermasz^1,5^, Wouter R. van Furth^1,2^

Affiliations: Center for Endocrine Tumors Leiden, Leiden University Medical Center, Leiden, The Netherlands^1^;

Department of Neurosurgery, Leiden University Medical Center, Leiden, The Netherlands^2^;

Department of Orthopaedics, Rehabilitation Medicine and Physical Therapy, Leiden University Medical Center, Leiden, The Netherlands^3^;

Medical Decision Making, Department of Biomedical Data Sciences, Leiden University Medical Center, Leiden, The Netherlands^4^;

Department of Medicine, Division of Endocrinology, Leiden University Medical Center, Leiden, The Netherlands^5^;

Department of Neurosurgery, Haaglanden Medical Center, The Hague, The Netherlands^6^

E-mail of Corresponding author: d.j.lobatto@lumc.nl

| **Supplementary table 2.** Unit prices in euros (€) | | | | |
| --- | --- | --- | --- | --- |
|  | ***Price (€)*** | ***Year*** | ***Source*** | ***Remark*** |
| Cost of surgery | 4551 | 2019 | Open DIS^1^ | Per operation |
| Inpatient care | 679 | 2019 | Guideline^2^ | Per day |
| Telephone consultation / e-consultation | 87 | 2019 | Guideline^3^ | Per consultation |
| Outpatient care | 173 | 2019 | Guideline^2^ | Per visit |
| Emergency care | 274 | 2019 | Guideline^2^ | Per visit |
| ^1^ National average costs for comparable medical treatments, excluding hospital days  ^2^ Dutch guidelines for healthcare cost calculation  ^3^ Consistent with GP consultations, specialist telephone consultations were valued at 50% of face-to-face consultations | | | | |
