# Supplementary material for: Feasibility, safety, and outcomes of a stratified fast-track care trajectory in pituitary surgery
Source: Endocrine. 2020 May 2;69(1):175–87. doi: 10.1007/s12020-020-02308-2 (PMC7343751; doi:10.1007/s12020-020-02308-2)
Supplement: Supplementary file 3 — Supplementary Table 3 [file 12020_2020_2308_MOESM3_ESM.docx]

Title: Feasibility, safety and outcomes of a stratified fast-track care trajectory in pituitary surgery

Journal: Endocrine

Authors: Daniel J. Lobatto^1,2^, Thea. P.M. Vliet Vlieland^1,3^, Wilbert B. van den Hout^1,4^, Friso de Vries^1,5^, Anne F. de Vries^1,2^, Pieter J. Schutte^1,2^, Marco J.T. Verstegen^1,2^, Alberto M. Pereira^1,5^, Wilco C. Peul^1,2,6^, Nienke R. Biermasz^1,5^, Wouter R. van Furth^1,2^

Affiliations: Center for Endocrine Tumors Leiden, Leiden University Medical Center, Leiden, The Netherlands^1^;

Department of Neurosurgery, Leiden University Medical Center, Leiden, The Netherlands^2^;

Department of Orthopaedics, Rehabilitation Medicine and Physical Therapy, Leiden University Medical Center, Leiden, The Netherlands^3^;

Medical Decision Making, Department of Biomedical Data Sciences, Leiden University Medical Center, Leiden, The Netherlands^4^;

Department of Medicine, Division of Endocrinology, Leiden University Medical Center, Leiden, The Netherlands^5^;

Department of Neurosurgery, Haaglanden Medical Center, The Hague, The Netherlands^6^

E-mail of Corresponding author: d.j.lobatto@lumc.nl

| **Supplementary** **Table 3**. Patient-reported outcomes before and 6 weeks after surgery among patients treated for a pituitary tumor (after August 2016) | | | | | | | | | | |
| --- | --- | --- | --- | --- | --- | --- | --- | --- | --- | --- |
|  | **Fast-track**  **(N=65/79)** | | **Non-fast-track**  **(N=42/78)** | | **p-value*** | **Total group**  **(N=107/155)** | | **Historic cohort**  **(N=16/313)** | | **p-value**** |
| LBNQ-pituitary index, mean (SE)^1^ |  |  |  |  |  |  |  |  |  |  |
| at baseline | 22.88 | (2.28) | 24.00 | (2.70) |  | 23.32 | (1.74) | 27.40 | (4.10) |  |
| after 6 weeks | 16.41 | (2.01) | 18.10 | (2.30) | .650 | 17.07 | (1.52) | 15.38 | (2.59) | .120 |
| SF-36 MCS, mean (SE)^2^ |  |  |  |  |  |  |  |  |  |  |
| at baseline | 44.33 | (1.53) | 43.36 | (1.99) |  | 43.95 | (1.21) | 43.75 | (3.93) |  |
| after 6 weeks | 47.51 | (1.38) | 48.55 | (1.51) | .384 | 47.92 | (1.03) | 49.93 | (2.33) | .356 |
| SF-36 PCS, mean (SE)^2^ |  |  |  |  |  |  |  |  |  |  |
| at baseline | 43.70 | (1.28) | 39.73 | (1.59) |  | 42.14 | (1.02) | 40.22 | (2.74) |  |
| after 6 weeks | 41.00 | (1.08) | 38.30 | (1.23) | .498 | 39.94 | (0.82) | 38.44 | (2.53) | .434 |
| EQ-index, mean (SE)^2^ |  |  |  |  |  |  |  |  |  |  |
| at baseline | 0.886 | (0.01) | 0.866 | (0.02) |  | 0.878 | (0.01) | 0.869 | (0.02) |  |
| after 6 weeks | 0.886 | (0.02) | 0.875 | (0.02) | .929 | 0.882 | (0.01) | 0.900 | (0.02) | .372 |
| EQ-VAS, mean (SE)^2^ |  |  |  |  |  |  |  |  |  |  |
| at baseline | 72.45 | (2.04) | 66.58 | (3.38) |  | 70.15 | (1.84) | 68.25 | (4.42) |  |
| after 6 weeks | 67.72 | (2.96) | 68.51 | (3.30) | .463 | 68.03 | (2.23) | 65.44 | (5.82) | .747 |
| ASK nasal-12, mean (SE)^2^ |  |  |  |  |  |  |  |  |  |  |
| at baseline | 0.80 | (0.11) | 0.58 | (0.08) |  | 0.71 | (0.07) | 0.60 | (0.16) |  |
| after 6 weeks | 1.10 | (0.12) | 0.97 | (0.12) | .926 | 0.94 | (0.19) | 0.94 | (0.19) | .778 |
| SNOT-22, mean (SE)^2^ |  |  |  |  |  |  |  |  |  |  |
| at baseline | 25.70 | (2.75) | 22.92 | (2.32) |  | 24.61 | (1.91) | 25.94 | (4.87) |  |
| after 6 weeks | 26.51 | (2.69) | 27.43 | (3.02) | .412 | 26.87 | (2.04) | 25.03 | (3.75) | .524 |
| VFQ-25, mean (SE)^2^ |  |  |  |  |  |  |  |  |  |  |
| at baseline | 84.37 | (1.94) | 76.63 | (2.75) |  | 81.33 | (1.64) | 84.01 | (3.22) |  |
| after 6 weeks | 91.08 | (1.27) | 86.16 | (1.65) | .095 | 89.15 | (1.04) | 91.33 | (1.88) | .545 |
| * fast-track versus non-fast-track (corrected for baseline), ** total versus historic cohort (corrected for baseline)  LBNQ-Pituitary (Leiden bother and needs questionnaire-pituitary), SF-36 (short form-36), MCS (mental component scale), PCS (physical component scale), EQ-5D (EuroQoL), VAS (visual analog scale), ASK-12 (anterior skullbase nasal inventory-12), SNOT-22 (sino-nasal outcome test-22), VFQ-25 (visual functioning questionnaire-25), SE (standard error)  ^1^ Higher scores indicate better HRQoL, health status, visual functioning and increased nasal morbidity  ^2^ Lower scores indicate lower disease burden  (bold) p<0.05 | | | | | | | | | | |
