# Supplementary material for: Feasibility, safety, and outcomes of a stratified fast-track care trajectory in pituitary surgery
Source: Endocrine. 2020 May 2;69(1):175–87. doi: 10.1007/s12020-020-02308-2 (PMC7343751; doi:10.1007/s12020-020-02308-2)
Supplement: Supplementary file 4 — Supplementary Table 4 [file 12020_2020_2308_MOESM4_ESM.docx]

Title: Feasibility, safety and outcomes of a stratified fast-track care trajectory in pituitary surgery

Journal: Endocrine

Authors: Daniel J. Lobatto^1,2^, Thea. P.M. Vliet Vlieland^1,3^, Wilbert B. van den Hout^1,4^, Friso de Vries^1,5^, Anne F. de Vries^1,2^, Pieter J. Schutte^1,2^, Marco J.T. Verstegen^1,2^, Alberto M. Pereira^1,5^, Wilco C. Peul^1,2,6^, Nienke R. Biermasz^1,5^, Wouter R. van Furth^1,2^

Affiliations: Center for Endocrine Tumors Leiden, Leiden University Medical Center, Leiden, The Netherlands^1^;

Department of Neurosurgery, Leiden University Medical Center, Leiden, The Netherlands^2^;

Department of Orthopaedics, Rehabilitation Medicine and Physical Therapy, Leiden University Medical Center, Leiden, The Netherlands^3^;

Medical Decision Making, Department of Biomedical Data Sciences, Leiden University Medical Center, Leiden, The Netherlands^4^;

Department of Medicine, Division of Endocrinology, Leiden University Medical Center, Leiden, The Netherlands^5^;

Department of Neurosurgery, Haaglanden Medical Center, The Hague, The Netherlands^6^

E-mail of Corresponding author: d.j.lobatto@lumc.nl

| **Supplementary table 4.** Baseline characteristics of patients with a pituitary tumor | | | | | | | |
| --- | --- | --- | --- | --- | --- | --- | --- |
|  | **Fast-track**  **(N=79)** | | | **Selection of historic cohort**  **(N=213)** | | | **P-value** |
| **Sociodemographic characteristics** |  | | |  | | |  |
| Female gender, N (%) | 43 (54.4) | | | 105 (49.3) | | | .510 |
| Age in years, mean (SD) | 47.2 (16.0) | | | 52.0 (16.7) | | | **.030** |
| Comorbidities, N (%) |  |  | |  | | |  |
| Diabetes mellitus | 3 (3.8) | | | 20 (9.4) | | | .145 |
| Neurovascular disease | 11 (13.9) | | | 26 (12.2) | | | .843 |
| Cardiovascular disease | 17 (21.5) | | | 69 (32.4) | | | .083 |
| Malignancies | 8 (10.1) | | | 22 (10.3) | | | 1.000 |
| Pulmonary disease | 1 (1.3) | | | 20 (9.4) | | | **.019** |
| Ophthalmologic disease | 15 (19.0) | | | 39 (18.3) | | | .867 |
| **Disease-specific characteristics** |  | |  | |  | |  |
| Tumor type, N (%) |  | | |  | | |  |
| NFA | 40 (50.6) | | | 119 (55.9) | | |  |
| ACRO | 15 (19.0) | | | 47 (22.1) | | |  |
| CD | 0 (0.0) | | | 0 (0.0) | | |  |
| PRL | 20 (25.3) | | | 29 (13.6) | | |  |
| RCC | 4 (5.1) | | | 13 (6.1) | | |  |
| Cranio | 0 (0.0) | | | 0 (0.0) | | |  |
| TSH-oma | 0 (0.0) | | | 5 (2.3) | | | .158 |
| Tumor size, N (%) |  |  | |  | |  |  |
| Micro | 19 (24.1) | | | 28 (13.1) | | |  |
| Macro | 60 (75.9) | | | 185 (86.9) | | |  |
| Giant | 0 (0.0) | | | 0 (0.0) | | | **.031** |
| Cavernous sinus invasion, N (%) | 12 (15.2) | | | 52 (24.5) | | | .152 |
| Time since diagnosis in years, median (IQR) | 1.1 (0.2-5.2) | | | 0.6 (0.1-3.8) | | | .199 |
| Prior treatments, N (%) |  |  | |  | |  |  |
| No treatment | 40 (50.6) | | | 127 (59.6) | | | .184 |
| Medication | 31 (39.2) | | | 61 (28.6) | | | .090 |
| Surgery | 11 (13.9) | | | 36 (16.9) | | | .595 |
| Radiotherapy | 0 (0.0) | | | 3 (1.4) | | | .566 |
| Apoplexy, N (%) | 4 (5.1) | | | 12 (5.6) | | | 1.000 |
| Preoperative endocrine status, N (%) |  |  | |  | |  |  |
| No deficits | 40 (50.6) | | | 108 (50.7) | | |  |
| Single hormone deficiency | 15 (19.0) | | | 27 (12.7) | | |  |
| Single hormone deficiency + DI | 0 (0.0) | | | 0 (0.0) | | |  |
| Multiple hormone deficiencies | 24 (30.4) | | | 78 (36.6) | | |  |
| Multiple hormone deficiencies + DI | 0 (0.0) | | | 0 (0.0) | | |  |
| DI alone | 0 (0.0) | | | 0 (0.0) | | | .327 |
| Preoperative visual status, N (%) |  |  | |  | |  |  |
| No deficits | 51 (64.6) | | | 106 (54.9) | | | .102 |
| Cranial nerve palsy, N (%) | 2 (2.5) | | | 4 (1.9) | | | .661 |
| N (number), SD (standard deviation), IQR (interquartile range), NFA (non-functioning pituitary adenoma), ACRO (acromegaly), CD (Cushing’s disease), PRL (prolactinoma), RCC (Rathke’s cleft cyst), Cranio (craniopharyngioma), TSH (thyroid-stimulating hormone), (bold) p<0.05  Due to rounding, not all percentages of the categorical variables add up to 100% | | | | | | | |
