# Supplementary material for: Feasibility, safety, and outcomes of a stratified fast-track care trajectory in pituitary surgery
Source: Endocrine. 2020 May 2;69(1):175–87. doi: 10.1007/s12020-020-02308-2 (PMC7343751; doi:10.1007/s12020-020-02308-2)
Supplement: Supplementary file 5 — Supplementary Table 5 [file 12020_2020_2308_MOESM5_ESM.docx]

Title: Feasibility, safety and outcomes of a stratified fast-track care trajectory in pituitary surgery

Journal: Endocrine

Authors: Daniel J. Lobatto^1,2^, Thea. P.M. Vliet Vlieland^1,3^, Wilbert B. van den Hout^1,4^, Friso de Vries^1,5^, Anne F. de Vries^1,2^, Pieter J. Schutte^1,2^, Marco J.T. Verstegen^1,2^, Alberto M. Pereira^1,5^, Wilco C. Peul^1,2,6^, Nienke R. Biermasz^1,5^, Wouter R. van Furth^1,2^

Affiliations: Center for Endocrine Tumors Leiden, Leiden University Medical Center, Leiden, The Netherlands^1^;

Department of Neurosurgery, Leiden University Medical Center, Leiden, The Netherlands^2^;

Department of Orthopaedics, Rehabilitation Medicine and Physical Therapy, Leiden University Medical Center, Leiden, The Netherlands^3^;

Medical Decision Making, Department of Biomedical Data Sciences, Leiden University Medical Center, Leiden, The Netherlands^4^;

Department of Medicine, Division of Endocrinology, Leiden University Medical Center, Leiden, The Netherlands^5^;

Department of Neurosurgery, Haaglanden Medical Center, The Hague, The Netherlands^6^

E-mail of Corresponding author: d.j.lobatto@lumc.nl

| **Supplementary table 5**. Surgical outcomes and costs among 292 surgically treated patients with a pituitary tumor stratified according to cohort | | | | | | | |
| --- | --- | --- | --- | --- | --- | --- | --- |
|  | **Fast-track**  **(N=79)** | | **Selection of historic cohort**  **(N=213)** | | | **p-value** | |
| Length of stay, mean (SD) | 3.0 (1.9) | | 5.1 (3.2) | | | **<.001** | |
| **Complications** |  |  | |  |  | |  |
| Number of readmitted patients, N (%) | 13 (16.5) | | 22 (10.3) | | | .160 | |
| Length of stay of all readmissions, mean (SD) | 3.6 (2.7) | | 4.5 (3.6) | | | .605 | |
| Any complication, N (%) | 38 (48.1) | | 118 (55.4) | | | .292 | |
| Transient DI, N (%) | 20 (25.3) | | 31 (14.6) | | | **.036** | |
| Permanent DI, N (%) | 3 (3.8) | | 11 (5.2) | | | .765 | |
| Delayed hyponatremia, N (%) | 9 (11.4) | | 24 (11.3) | | | 1.000 | |
| New onset pituitary deficiency, N (%) | 4 (5.1) | | 18 (8.5) | | | .456 | |
| Postoperative CSF leak, N (%) | 3 (3.8) | | 15 (7.1) | | | .416 | |
| Epistaxis, N (%) | 10 (12.7) | | 22 (10.3) | | | .536 | |
| Postoperative intracranial haemorrhage, N (%) | 0 (0.0) | | 1 (0.5) | | | 1.000 | |
| **Hospital costs (in euro’s)** |  |  | |  |  | |  |
| Admission, mean (SD) | 7249 (1318) | | 8660 (2203) | | | **<0.001** | |
| Readmission, mean (SD) | 438 (1221) | | 338 (1256) | | | .541 | |
| Emergency room visits, mean (SD) | 42 (108) | | 26 (84) | | | .188 | |
| Outpatient clinic visits, mean (SD) | 55 (102) | | 346 (0) | | | **<0.001** | |
| E-mail contacts, mean (SD) | 626 (284) | | 0 (0) | | | **<0.001** | |
| Telephone contacts, mean (SD) | 242 (184) | | 0 (0) | | | **<0.001** | |
| Total hospital costs, mean (SD) | 8652 (1748) | | 9266 (2540) | | | **.021** | |
| N (number), SD (standard deviation), IQR (interquartile range), DI (diabetes insipidus), CSF (cerebrospinal fluid)  (bold) p<0.05 | | | | | | | |
